# Sodium quantification in skeletal muscle: comparison between Cartesian gradient-echo and radial ultra-short echo time ^23^Na MRI techniques

**DOI:** 10.1186/s41747-024-00461-1

**Published:** 2024-05-22

**Authors:** Teresa Gerhalter, Felix Schilling, Nour Zeitouni, Peter Linz, Pierre-Yves Baudin, Dennis Kannenkeril, Christoph Kopp, Anke Dahlmann, Roland Schmieder, Michael Uder, Armin M. Nagel, Lena V. Gast

**Affiliations:** 1 Institute of Radiology, University Hospital Erlangen, Friedrich-Alexander-University Erlangen-Nürnberg (FAU), Maximiliansplatz 3, 91054 Erlangen, Germany; 2 Department of Nephrology and Hypertension, Friedrich-Alexander-University Erlangen-Nürnberg (FAU), Erlangen, Germany; 3 NMR laboratory, Neuromuscular Investigation Center, Institute of Myology, Paris, France; 4 Division of Medical Physics in Radiology, German Cancer Research Centre (DKFZ), Heidelberg, Germany

**Keywords:** Calibration, Magnetic resonance imaging, Muscle (skeletal), Sodium, Volunteers

## Abstract

**Background:**

Clinical magnetic resonance imaging (MRI) studies often use Cartesian gradient-echo (GRE) sequences with ~2-ms echo times (TEs) to monitor apparent total sodium concentration (aTSC). We compared Cartesian GRE and ultra-short echo time three-dimensional (3D) radial-readout sequences for measuring skeletal muscle aTSC.

**Methods:**

We retrospectively evaluated 211 datasets from 112 volunteers aged 62.3 ± 12.1 years (mean ± standard deviation), acquired at 3 T from the lower leg. For ^23^Na MRI acquisitions, we used a two-dimensional Cartesian GRE sequence and a density-adapted 3D radial readout sequence with cuboid field-of-view (DA-3D-RAD-C). We calibrated the ^23^Na MR signal using reference tubes either with or without agarose and subsequently performed a relaxation correction. Additionally, we employed a six-echo ^1^H GRE sequence and a multi-echo spin-echo sequence to calculate proton density fat fraction (PDFF) and water T2. Paired Wilcoxon signed-rank test, Cohen *d*_*z*_ for paired samples, and Spearman correlation were used.

**Results:**

Relaxation correction effectively reduced the differences in muscle aTSC between the two acquisition and calibration methods (DA-3D-RAD-C using NaCl/agarose references: 20.05 *versus* 19.14 mM; *d*_*z*_ = 0.395; Cartesian GRE using NaCl/agarose references: 19.50 *versus* 18.82 mM; *d*_*z*_ = 0.427).

Both aTSC of the DA-3D-RAD-C and Cartesian GRE acquisitions showed a small but significant correlation with PDFF as well as with water T2.

**Conclusions:**

Different ^23^Na MRI acquisition and calibration approaches affect aTSC values. Applying relaxation correction is advised to minimize the impact of sequence parameters on quantification, and considering additional fat correction is advisable for patients with increased fat fractions.

**Relevance statement:**

This study highlights relaxation correction’s role in improving sodium MRI accuracy, paving the way for better disease assessment and comparability of measured sodium signal in patients.

**Key points:**

• Differences in MRI acquisition methods hamper the comparability of sodium MRI measurements.

• Measured sodium values depend on used MRI sequences and calibration method.

• Relaxation correction during postprocessing mitigates these discrepancies.

• Thus, relaxation correction enhances accuracy of sodium MRI, aiding its clinical use.

**Graphical Abstract:**

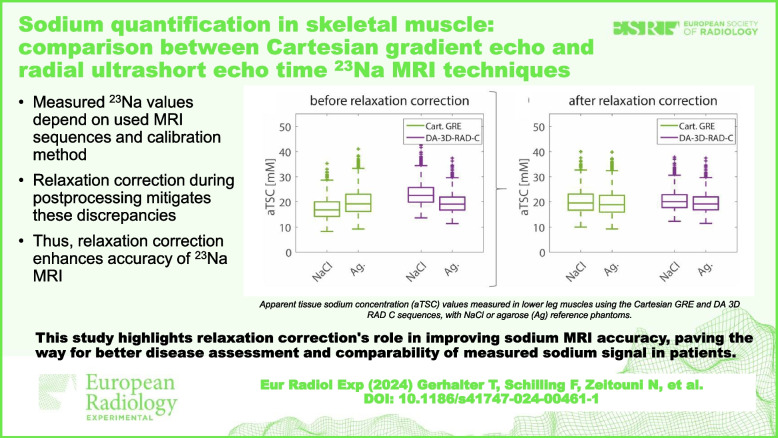

**Supplementary Information:**

The online version contains supplementary material available at 10.1186/s41747-024-00461-1.

## Background

Sodium (^23^Na) magnetic resonance imaging (MRI) has gained increasing use as a method to evaluate sodium homeostasis within skeletal muscle tissue [1]. Its successful application spans various pathologies, detecting changes in muscular sodium content, from (neuro-)muscular diseases [2, 3] to renal impairment [4–6] and cardiovascular diseases [7, 8]. Several studies have highlighted its reproducibility in determining sodium concentrations in skeletal muscle tissue [9, 10]. Despite these advancements, there remains a lack of standardization in both acquisition and quantification methods for ^23^Na MRI. Establishing such standards is crucial to facilitate meaningful comparisons and widespread dissemination of the technique.

Typically, ^23^Na MRI data is used to determine the apparent tissue sodium concentration (aTSC), representing an average of intracellular and extracellular sodium concentrations, weighted by the respective volume fractions of these spaces. Achieving a spin-density weighted acquisition is essential to obtain contrast solely reflecting tissue sodium content. This involves using a measurement with long repetition time (TR) and short echo time (TE): TR longer than five T1 and TE much shorter than T2^*^ [1].

In biological tissues such as the muscle, ^23^Na nuclei exhibit a biexponential signal decay with a short component, T2_s_^*^, and a long component, T2_l_^*^:

1 $${S}_{23Na}={M}_{0}\left(0.6 {\text{exp}}\left(-\frac{TE}{{T2}_{s}^{*}}\right)+0.4 {\text{exp}}\left(-\frac{TE}{{T2}_{l}^{*}}\right)\right)$$

Within skeletal muscle tissue, T2_s_^*^ ranges from 0.6 to 3.6 ms, while T2_l_^*^ has been measured in the range of 11–26 ms [1]. Given that the short component constitutes 60% of the total ^23^Na signal, specific pulse sequences are necessary to capture the rapidly decaying ^23^Na signal and achieve a spin-density weighted image contrast. Typically, radial or spiral schemes starting the acquisition in the center of k-space, enabling ultra-short echo times (UTE), are used for quantitative ^23^Na MRI [11]. However, besides optimized UTE sequence for ^23^Na MRI, Cartesian gradient-echo (GRE) sequences with TE of about 2 ms are still frequently used in clinical studies to monitor changes in the aTSC in skeletal muscle tissue (*e.g.*, [5, 12]; summary in Additional file 1: Supplemental Tables S1 and S2).

Theoretically, ^23^Na nuclei in muscle tissue undergo also a biexponential longitudinal relaxation. However, a separation of the long and short T1 components is challenging due to the 20/80% ratio for the short and long components of T1 as well as due to the smaller difference between them, compared to the larger difference between the T2 components [13]. Typical monoexponential ^23^Na T1 values are in the range of 25–33 ms for skeletal muscle tissue and around 60 ms for saline solutions [1]. These T1 values are crucial for quantitative ^23^Na imaging, where reference compartments with known sodium concentrations are required for calibration of the ^23^Na MR signal. For imaging of skeletal muscle tissue, external reference phantoms filled with NaCl solution or agarose gels are typically used for this purpose. While agarose gel has the advantage of relaxation behavior more similar to biological tissues, it can also mold over time and therefore needs more careful preparation. Conversely, using stable NaCl solutions may introduce quantification biases if the TR is excessively short compared to the solutions’ T1. To reduce the influence of different relaxation times, relaxation effects can be corrected by introducing relaxation correction factors as done also in previous ^23^Na MRI studies on muscle [10, 14–16].

In this study, we conducted a retrospective comparison of sodium quantification using a Cartesian GRE sequence against a density-adapted three-dimensional (3D) radial readout sequence with cuboid field-of-view (DA-3D-RAD-C) [17]. Initially, we measured the aTSC of leg muscles for both sequences based on external references: agarose-filled and NaCl solution without additional agarose filling. Additionally, we hypothesized that correcting for relaxation biases, introduced by the acquisition parameters and relaxation properties of the external references used, would reduce differences in measured aTSC, enhancing comparability among clinical studies utilizing ^23^Na MRI in skeletal muscle tissue.

## Methods

### Subjects and study design

For this retrospective study, we evaluated 211 data sets of 112 subjects including (79 males), aged 68.3 ± 11.8 years (mean ± standard deviation), ranging 21−86 years, with or without known cardiovascular/renal diseases, which were examined in the context of different ongoing (NCT03128528, NCT06056466) or completed MRI studies [8]. Table 1 provides an overview of the evaluated datasets. We included subjects from four different cohorts: two cohorts from patients with known disorders that are suspected to show changes in the apparent tissue sodium concentration (aTSC) of muscle tissue and two cohorts, which are expected to be healthy as they were either kidney donors or invited as healthy controls. As for the patient cohorts, chronic heart failure patients were examined before and after treatment with empagliflozin [8] and Fontan patients before and after treatment with tolvaptan [18]. Data were acquired from August 2018 to August 2022 based on the approval of the local ethical review board (Number: *3948, 271_17B, 77_19 B and 77_17 Az*), and written informed consent was obtained before each exam. One data set was defined by containing data acquired from one scan session of a subject.

**Table 1:** Overview of subjects included in current data evaluation

**Subject description**	**Number of subjects**	**Mean age (years)**	**Males/females**	**Total number of data sets**	**Data sets including T2 mapping**
Chronic heart failure	74	66.45 ± 8.7 [45−86]	60/14	164	162
Fontan patients	3	41.5 ± 14.7 [21−55]	1/2	8	6
Kidney donors	23	53.6 ± 10.5 [30−71]	7/16	27	13
Healthy controls	12	63.1 ± 7.2 [49−91]	11/1	12	12
Total	112	62.3 ± 12.1 [21−86]	79/33	211	193

Age is presented as mean ± standard deviation [range]. We included subjects from four different cohorts to reach a total number of data sets of 211. Some data sets showed missing T2 mapping data, which is indicated in a separate column

Our data analysis inclusion criteria comprised the following:

i. Complete ^23^Na MRI data sets acquired using both the Cartesian GRE and DA-3D-RAD-C sequences;
ii. Measurements performed with the same reference phantom set-up;
iii. Dixon-type ^1^H MRI acquisition performed in same leg position.

Notably, some subjects underwent multiple MRI examinations, such as pre- and post-treatment, which was anticipated in the design of the initial MRI study (Table 1).

### MRI acquisition protocol and data processing

**Fig. 1:**
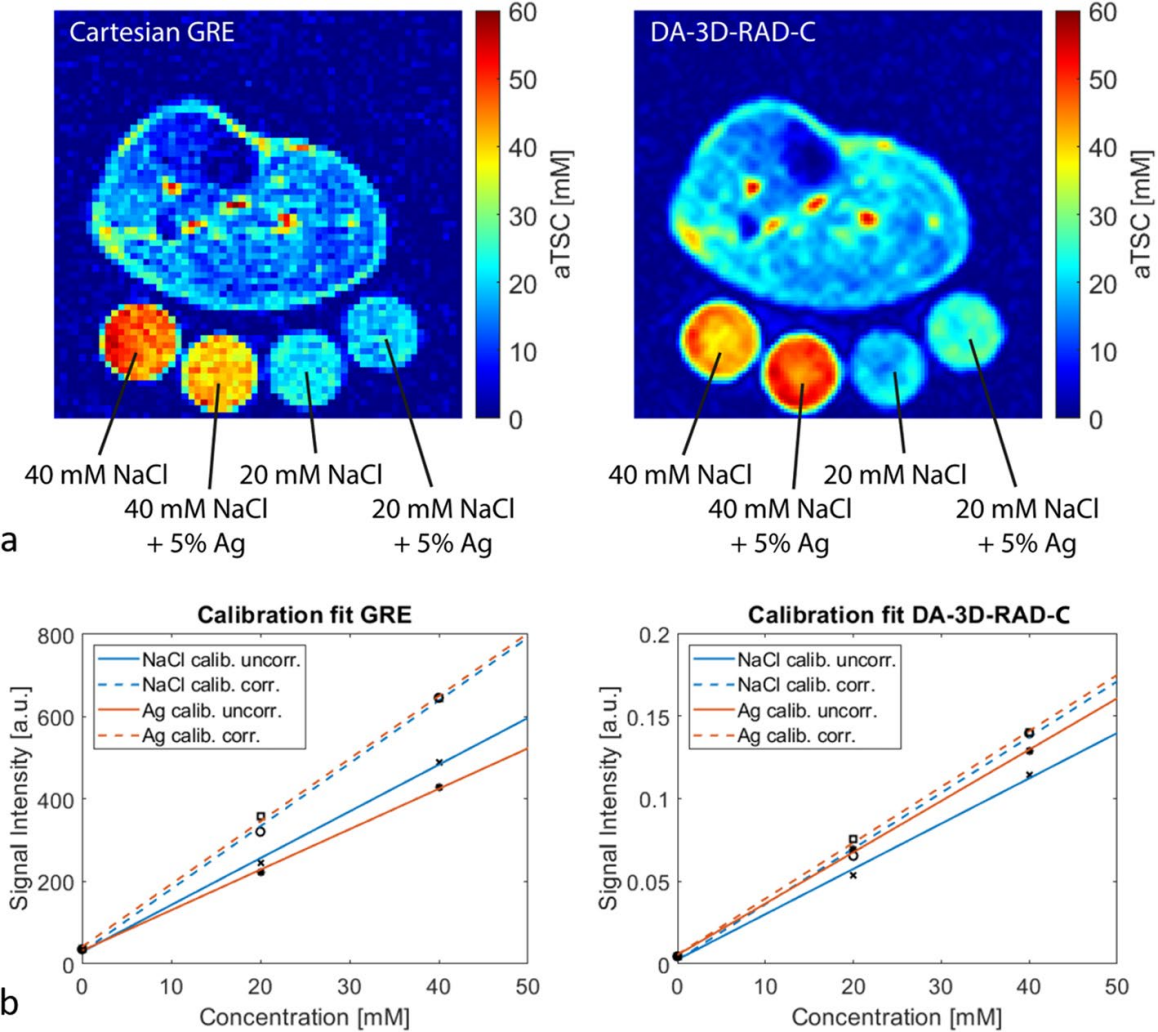
**a** Exemplary apparent tissue sodium concentration (aTSC) maps of the lower leg acquired with a Cartesian gradient-echo (GRE) and density-adapted 3D radial readout sequence with cuboid field-of-view (DA-3D-RAD-C) sequence (acquisition parameters presented in Table 1). Note that these are uncorrected aTSC maps using the agar references for calibration. DA-3D-RAD-C ^23^Na images were reconstructed using a Hamming filter. Four different reference tubes were scanned together with the lower leg and used for signal calibration. Note the differences in the signal intensities between the reference tubes with and without agarose (Ag). **b** Exemplary calibration curves for the Cartesian GRE and DA-3D-RAD-C images using either the reference phantoms without (blue) or with (orange) agarose. Before correcting relaxation weighting effects, there is a noticeable difference in the calibration curves based on NaCl and agarose phantoms, which can be reduced by applying a relaxation correction (dashed lines)

MRI data were acquired on two 3-T whole-body MRI system (Magnetom Skyra or VIDA, Siemens Healthineers, Erlangen, Germany) using a 1-channel transmit/receive birdcage knee radiofrequency coil (Stark Contrast, Erlangen, Germany) for ^23^Na MRI acquisition as well as the whole-body radiofrequency coil of the scanner for ^1^H imaging. The lower leg was positioned on a reference phantom holder, containing four tubes (35-mm diameter) filled with reference solutions containing two different NaCl concentrations (20 mM and 40 mM). Two of the tubes were filled with pure NaCl solution, and two were additionally containing 5% of agarose gel (Fig. 1). Imaging of ^23^Na and ^1^H was performed in the same position of the leg without the need of repositioning the leg.

For quantitative ^23^Na MRI acquisitions, two different sequences were used: a two-dimensional Cartesian GRE sequence with a TE of 2.07 ms and a DA-3D-RAD-C sequence with a TE of 0.3 ms. Acquisition parameters of the Cartesian GRE and DA-3D-RAD-C sequence for aTSC quantification are listed in Table 2. Both sequences were acquired at the isocenter of the magnet and had the same nominal spatial in-plane resolution and a similar total acquisition duration. Parts of the Cartesian ^23^Na GRE data have been published before [8, 18].

**Table 2:** Acquisition parameters for ^23^Na and ^1^H imaging at 3 T

**Acquisition parameters**	^**23**^**Na MRI**		^**1**^**H MRI**	
Sequence	2D Cartesian GRE	DA-3D-RAD-C	MESE	Dixon
Echo time (ms)	2.07	0.3	9.5-304 (32 equidistant echoes)	1.52, 3.07, 4.88, 6.71, 8.54, 10.37
Repetition time (ms)	100	100	3,000	50
Nominal FA (°)	90	90	90, 180	6
Nominal spatial resolution (mm^3^)	3 × 3 × 30	3 × 3 × 20	1.5 × 1.5 × 10	1.5 × 1.5 × 5
Field of view (mm)	192 × 192	192 × 192	192 × 192	192 × 192
Readout duration (ms)	2.33	10.0	2.22	1.25
Projections	-	6,152	-	-
Averages	128	1	1	1
Number of slices	1	-	5	36
Acquisition time (min:s)	13:41	10:15	6:29	4:02

*FA*, Flip angle; *MESE*, Multi-echo spin-echo sequence. ^23^Na signals were acquired with a 2D Cartesian gradient-echo sequence (GRE) and a 3D-density-adapted 3D radial sequence (DA-3D-RAD-C)

The ^1^H MRI protocol consisted of a 3D six-echo GRE sequence and a multi-echo spin-echo sequence (MESE). Proton density fat fraction (PDFF) maps were calculated automatically by the scanner from the GRE acquisition by a vendor-provided working package. MESE data were fitted using a triexponential model implemented in Python in each pixel to compensate for increased fat fraction of muscle tissue and extract water T2 [19]. For 18 data sets, MESE data was missing as the multi-echo images were not stored and thus not available for data treatment and analysis (Table 1).

To quantitatively assess the MRI data, regions of interest (ROIs) were semiautomatically delineated on concurrently acquired ^1^H-Dixon images using the Deep Anatomical Federated Network−DAFNE [20]. The analysis involved seven muscle ROIs: gastrocnemius medialis and lateralis, soleus, tibialis anterior and posterior, peroneus, and extensor digitorum longus. The same muscle ROIs were used for assessing PDFF, water T2, and aTSC maps. Moreover, binary masks for the four reference phantoms were manually segmented on the ^1^H-Dixon using the Medical Imaging Interaction Toolkit−MITK [21], cropped by one voxel (diameter 32 mm) and then co-registered to each ^23^Na data set (Additional file 1: Supplemental Fig. S1).

Reconstruction of the ^23^Na MRI data was performed offline using a custom-written MATLAB tool based on a non-uniform fast Fourier transform−NUFFT. A Hamming filter was applied to reduce Gibb’s ringing artifacts. Calibration of the ^23^Na signal was performed using a linear regression based on two sets of reference phantoms: (i) the mean value of the reference tubes containing 20 and 40 mM NaCl with 5% agarose, along with the standard deviation of the background signal, and (ii) the mean value of the reference tubes containing 20 and 40 mM NaCl without agarose, along with the standard deviation of the background signal. The standard deviation of the background signal was chosen for the calibration curve, as it aligns more with the standard approach for random noise measurements on magnitude images [22].

In the ^23^Na MRI protocol, slight T1 weighting (*i.e.*, TR < 5 T1) was accepted to reduce the total acquisition time. For the GRE acquisition, TE was in the range of the short transverse relaxation time (T2_s_^*^) of muscle tissue and agarose reference phantoms, leading to an additional slight T2^*^ weighting of the resulting images. To correct the measured aTSC for these relaxation biases, a relaxation correction was introduced for the reference phantoms as well as for muscle tissue as an additional postprocessing step before the signal calibration [10].

First, ^23^Na relaxometry was performed in 14 healthy volunteers (7 males and 7 females, aged 36.9 ± 8.1 years [mean ± standard deviation], ranging 23−49 years) using stack-of-stars sequences with multiple echoes for T2^*^ mapping and different repetition times for T1 mapping (Additional file 1: Supplemental material, Supplemental Table S3 for acquisition parameters and relaxation times calculations). The individual relaxation times were fitted over the mean intensity of ROIs for muscle and reference tubes and the average of these individual relaxation times was then used for relaxation time correction (Table 3).

**Table 3:** ^23^Na relaxation times used to estimate relaxation biases for reference phantoms and muscle tissue

	T1 (ms)	T2_s_^*^ (ms)	T2_l_^*^ (ms)	*c*_relax, DA-3D-RAD-C_	*c*_relax, GRE_
NaCl solution (20 mM)	56.4 ± 2.0	−	24.2 ± 6.4	0.8200	0.7621
NaCl solution (40 mM)	56.4 ± 2.0	−	22.8 ± 6.0	0.8193	0.7581
Agarose gel (5%, 20 mM)	26.8 ± 0.5	2.9 ± 0.2	11.7 ± 2.4	0.9086	0.6139
Agarose gel (5%, 40 mM)	26.8 ± 0.5	3.5 ± 0.3	15.2 ± 2.8	0.9203	0.6649
Muscle tissue	27.1 ± 2.0	3.5 ± 0.5	32.2 ± 3.2	0.9234	0.6896

T2^*^ and T1 values of muscle tissue were measured in 14 healthy subjects, aged 36.9 ± 8.1 years (mean ± standard deviation). Relaxation times are presented as mean ± standard deviation. Resulting relaxation correction factors *c*_relax, DA-3D-RAD-C_ for the DA-3D-RAD-C and *c*_relax, GRE_ gradient-echo acquisition were calculated based on Eq. 2 (NaCl solution) and Eq. 3 (agarose gel, muscle tissue). *c*_*relax, DA-3D-RAD-C*_, relaxation correction factor for DA-3D-RAD-C; *c*_*relax, GRE*_, relaxation correction factor for Cartesian gradient-echo; *NaCl* Sodium chloride

Based on the measured relaxation times and applied TE and TR, the signal values of the ^23^Na images were corrected with a monoexponential or biexponential relaxation correction factor, which was derived from the fast low-angle shot equation [1, 16]. For NaCl solution, a monoexponential signal behavior for T1 and T2^*^ was assumed to calculate the relaxation correction factors:

2 $${c}_{{\text{relax}},{\text{solution}}}=\left(1-{\text{exp}}\left(-\frac{TR}{T1}\right)\right){\text{exp}}\left(-\frac{TE}{{T2}^{*}}\right)$$

For muscle tissue and agarose gels, a monoexponential T1 and biexponential T2^*^ model was applied:

3 $${c}_{{\text{relax}},{\text{tissue}}}=\left(1-{\text{exp}}\left(-\frac{TR}{T1}\right)\right)\left(0.6 {\text{exp}}\left(-\frac{TE}{{T2}_{s}^{*}}\right)+0.4 {\text{exp}}\left(-\frac{TE}{{T2}_{l}^{*}}\right)\right),$$

assuming a short component fraction corresponding to the theoretical value of 60%. Resulting relaxation correction factors for the DA-3D-RAD-C and GRE acquisitions are summarized in Table 3. For relaxation correction, the uncorrected average ^23^Na signal from delineated muscle and reference ROIs were divided by the corresponding relaxation correction factor. Subsequently, the corrected muscle ^23^Na signals were calibrated using the linear regression approach based on the different reference tubes as described above. The fitting of relaxation times as well as calculation of relaxation correction factors and application of relaxation correction and signal calibration was performed in MATLAB using in-house written scripts.

The datasets analyzed during the current study are available from the corresponding author on reasonable request.

### Statistical analysis

Due to some measures not conforming to a normal distribution based on a Shapiro-Wilk test, results were presented using parametric [mean ± standard deviation] and non-parametric [median (interquartile range)] descriptive statistics.

Differences in aTSC between imaging acquisition methods and calibration approaches were assessed using the paired Wilcoxon signed-rank test (WSRT) for non-normally distributed samples. Cohen’s *d*_*z*_ for paired samples was employed to evaluate the impact of relaxation correction on aTSC values. Furthermore, relaxation-corrected aTSC values calibrated with agarose phantoms were compared across different muscles using a Kruskal-Wallis test. To explore the differences in aTSC between individual muscles, a multiple comparison test was conducted utilizing Tukey’s honestly significant difference (Tukey-HSD) procedure.

Furthermore, the comparison between Cartesian GRE and DA-3D-RAD-C data involved Spearman rank correlation analysis. Spearman rank correlations were also utilized to examine associations between aTSC and water T2 relaxation times as well as PDFF measured by ^1^H MRI.

All statistical tests were performed in SPSS (IBM SPSS statistics, version 28.0). A *p*-value < 0.05 was considered significant for all statistical tests.

## Results

In Fig. 1, we show exemplary aTSC maps resulting from both Cartesian GRE and DA-3D-RAD-C ^23^Na MRI data of the lower leg of a single volunteer, along with the corresponding calibration curves using either the reference phantoms with or without agarose. Differences between the signal calibration curves, calculated based on the NaCl and agarose phantoms, were reduced following the described correction for relaxation biases.

**Fig. 2:**
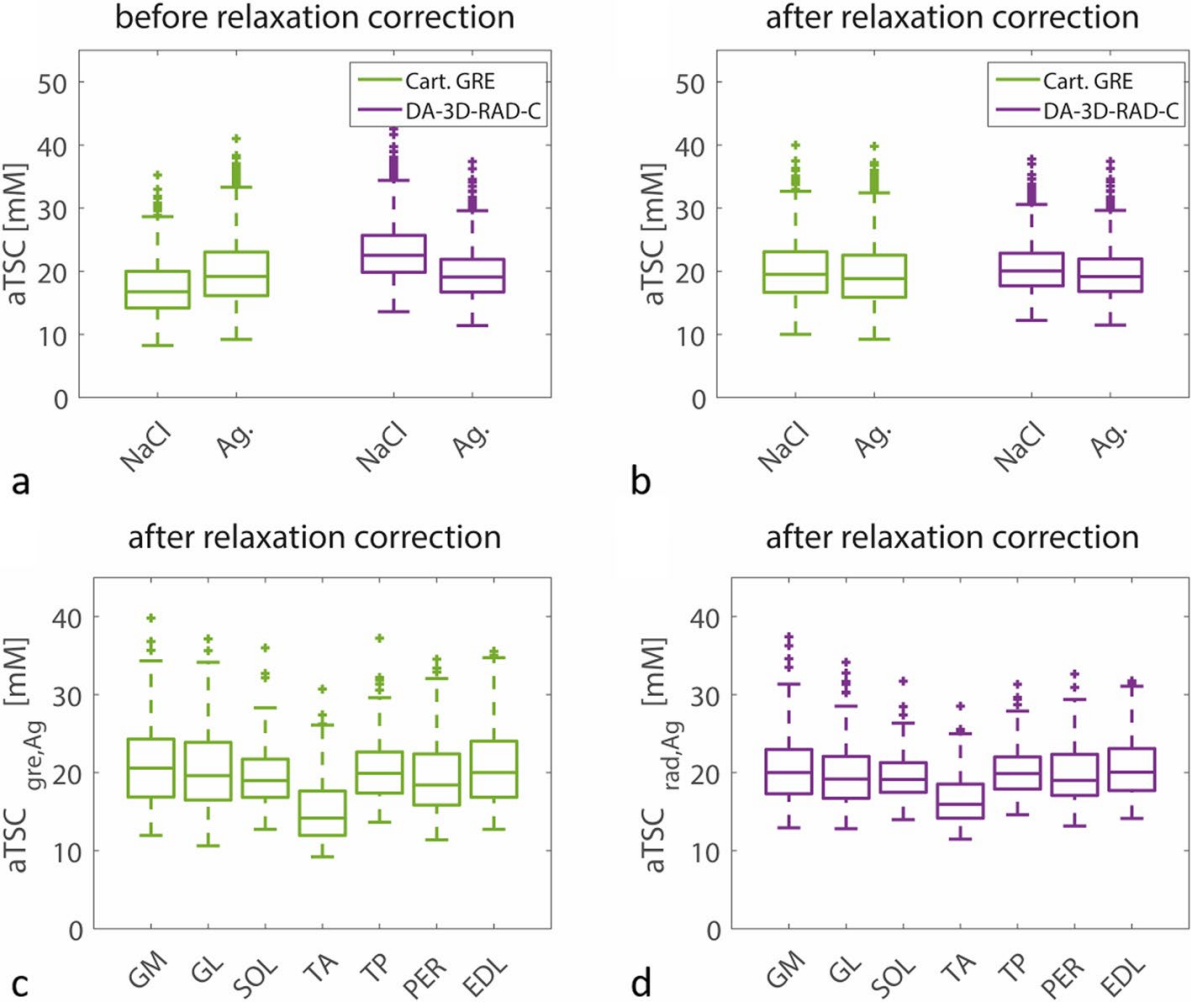
Comparison of apparent tissue sodium concentration (aTSC) values measured in lower leg muscles (*n* = 1,491) using the Cartesian gradient-echo (GRE) and DA-3D-RAD-C sequences and calibrating the ^23^Na MR signal either using NaCl and agarose (Ag) reference phantoms. **a** Without performing a relaxation correction, aTSC values differed between the two sequences and reference types. The difference between agarose and NaCl calibration was smaller for the DA-3D-RAD-C (Cohen *d*_*z*_ = 0.686) than for the Cartesian GRE acquisition (Cohen *d*_*z*_ = 0.910). **b** The correction of relaxation biases reduced the difference in measured aTSC values for the DA-3D-RAD-C (Cohen *d*_*z*_ = 0.395) and for the Cartesian GRE acquisition (Cohen *d*_*z*_ = 0.427). **c**, **d** aTSC values in TA muscles (*n* = 213) were significantly decreased compared to all other lower leg muscle regions for both sequence types (*p* < 0.001). Furthermore, aTSC values of the Cartesian GRE were reduced in the PER muscle compared to the GM and EDL but not for the DA-3D-RAD-C sequence. *DA-3D-RAD-C*, Density-adapted 3D radial readout sequence with cuboid field-of-view; *EDL*, Extensor digitorum longus; *GM*, Gastrocnemius medialis; *GL*, Gastrocnemius lateralis; *PER*, Peroneus; *SOL*, Soleus; *TA*, Tibialis anterior; *TP*, Tibialis posterior

Mean aTSC values were measured in seven different muscle regions for all 213 data sets. Figure 2 shows the comparison of resulting aTSC values between the two sequence and reference types before and after correction of relaxation biases. The aTSC for the DA-3D-RAD-C images was significantly lower when agarose references were used for calibration (median 22.5 [interquartile range 5.8] mM *versus* 19.1 [5.2] mM, WSRT *p* < 0.001; Cohen *d*_*z*_ = 0.686). The opposite was for Cartesian GRE imaging (median 16.7 [5.8] mM *versus* 19.2 [6.9] mM, WSRT *p* < 0.001; Cohen *d*_*z*_ = 0.91) in case no relaxation correction was performed. Cartesian GRE imaging yielded lower aTSC values than DA-3D-RAD-C acquisition. After relaxation correction, the values were similar for both sequences and reference types (Additional file 1: Supplemental Table S4), which is also reflected by the reduced effect sizes for the DA-3D-RAD-C (Cohen *d*_*z*_ = 0.395) and for the Cartesian GRE acquisition (Cohen *d*_*z*_ = 0.427).

When examining specific muscles in the lower leg, the tibialis anterior consistently displayed notably lower aTSC values compared to all other lower leg muscle regions, regardless of the sequence or reference phantom types utilized (Fig. 2c, d, *p* < 0.001, Tukey-HSD). Additionally, in Cartesian GRE imaging, the peroneus exhibited smaller aTSC values in comparison to the extensor digitorum longus (*p* < 0.048, Tukey-HSD) and gastrocnemius medialis (*p* < 0.020, Tukey-HSD), whereas this distinction was not evident in DA-3D-RAD-C acquisitions.

**Fig. 3:**
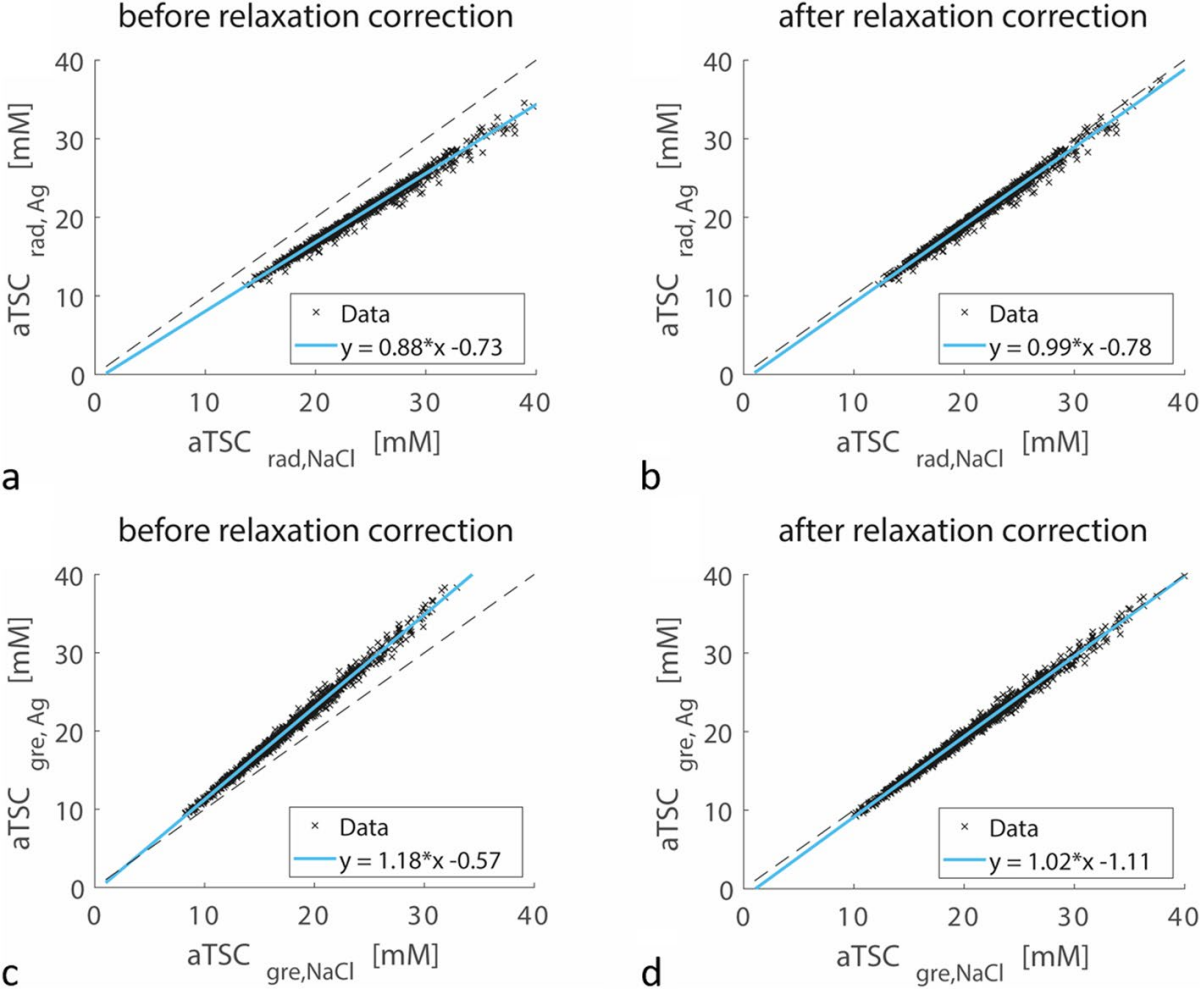
Correlation between apparent tissue sodium concentration (aTSC) values measured in lower leg muscles (*n* = 1,491) using the density-adapted 3D radial readout sequence with cuboid field-of-view (DA-3D-RAD-C) (**a**, **b**) and Cartesian gradient-echo (GRE) (**c**, **d**) sequence by calibrating the signal intensity either using the agarose (Ag) or NaCl reference phantoms. The dashed lines represent perfect correlation. If no correction for relaxation effects was performed (**a**, **c**), the measured aTSC values differed between the two reference phantom types (**a** Spearman rho = 0.995; **c** Spearman rho = 0.997). After relaxation correction (**b**, **d**), data was aligned with the dashed line (**b** Spearman rho = 0.995; **d** Spearman rho = 0.997)

The influence of the selected reference phantom types on the measured aTSC values is detailed in Fig. 3. Prior to applying relaxation corrections, normalizing on NaCl phantoms led to higher aTSC values for the DA-3D-RAD-C data, whereas lower aTSC values were observed for the Cartesian GRE data when compared to normalization with agarose phantoms. After relaxation correction, aTSC values between the two reference phantom types showed strong correlation, exhibiting a fitted linear slope close to one for both sequence types.

**Fig. 4:**
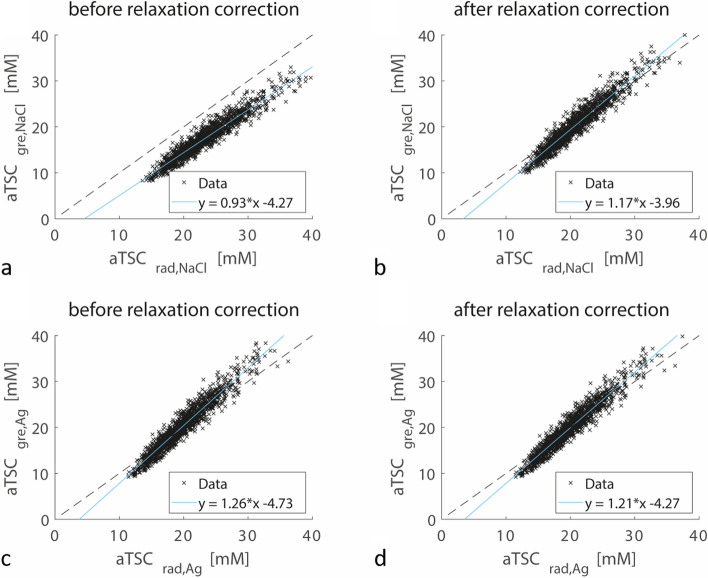
Correlation of apparent tissue sodium concentration (aTSC) values in lower leg muscles (*n* = 1,491) measured using the density-adapted 3D radial readout with cuboid field-of-view (DA-3D-RAD-C) sequence and the Cartesian gradient-echo (GRE) sequence by calibrating the signal intensity either using the agarose (Ag) (**a**, **b**) or NaCl reference phantoms (**c**, **d**). The dashed lines represent perfect correlation. Strong dependencies were found between each correlation with Spearman rho coefficients for panels (**a**) 0.955, (**b**) 0.954, (**c**) 0.959, and (**d**) 0.959

Likewise, Fig. 4 demonstrates a high correlation between aTSC values measured with DA-3D-RAD-C and Cartesian GRE sequences. When signal calibration was performed based on NaCl phantoms, aTSC values measured by Cartesian GRE were generally lower compared to DA-3D-RAD-C. However, even after relaxation effect correction, the slope of the fitted correlation curves between DA-3D-RAD-C and Cartesian GRE remained greater than one (1.17 for NaCl phantom calibration, 1.21 for agarose phantom calibration).

**Fig. 5:**
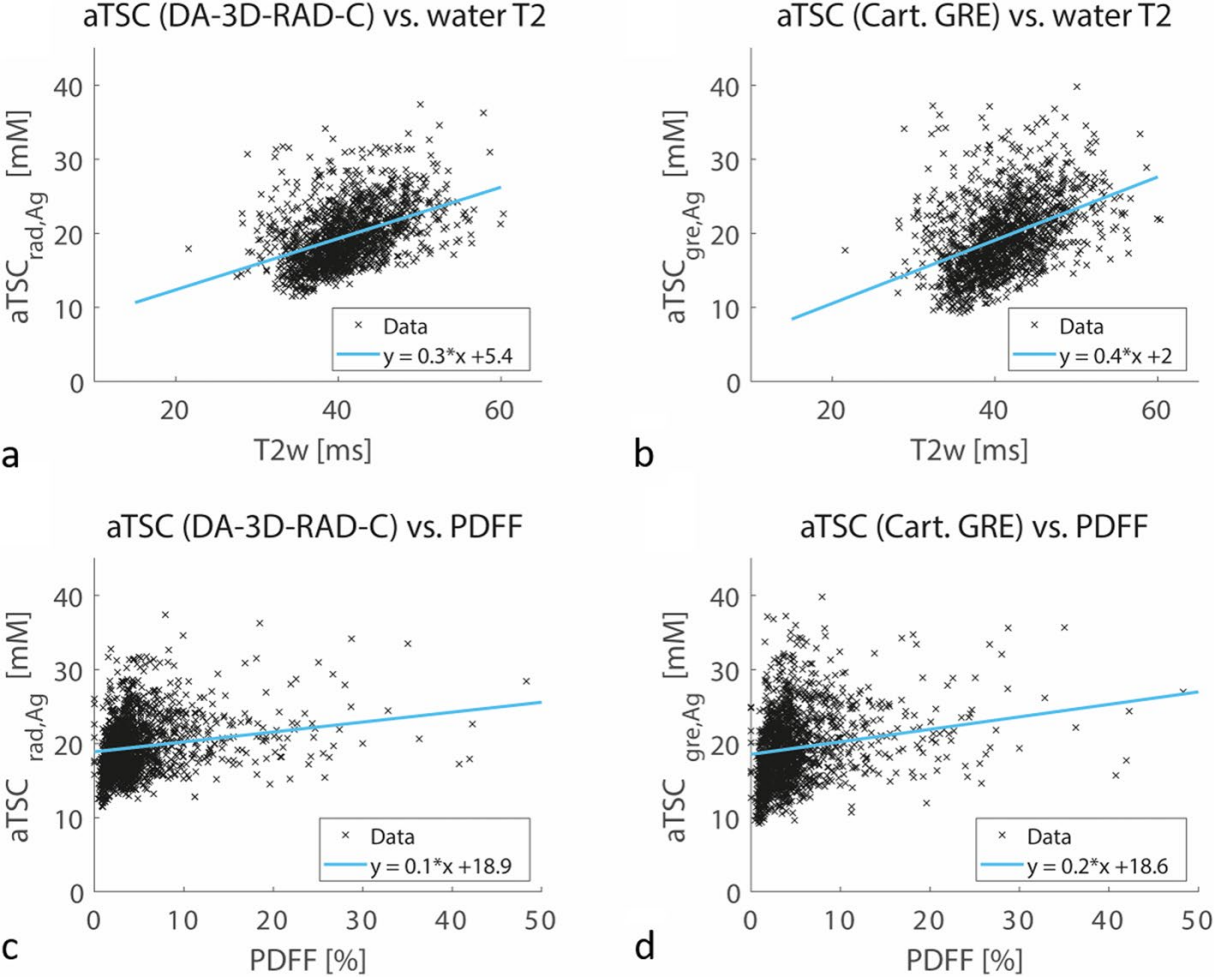
Correlations between apparent tissue sodium concentration (aTSC) (including relaxation correction, normalized on agarose phantoms) and water T2 relaxation times (**a**, **b**) and proton-density fat fraction (PDFF) (**c**, **d**) measured by ^1^H MRI. Note the positive, but not perfect correlation between aTSC and water T2:_:_ DA-3D-RAD-C, Spearman rho = 0.472, *p* < 0.001; Cartesian GRE, Spearman rho = 0.467, *p* < .001). The PDFF is less dependent on the aTSC; DA-3D-RAD-C, Spearman rho = 0.357, *p* < 0.001; Cartesian GRE, Spearman rho = 0.359, *p* < 0.001. *DA-3D-RAD-C* Density-adapted 3D radial readout with cuboid field-of-view, *GRE* Gradient-echo

To explore potential sources for deviations in both low and high aTSC ranges, correlations were examined between relaxation-corrected aTSC, calibrated with agarose phantoms, and water T2 along with PDFF measured by ^1^H MRI (Fig. 5). The average PDFF of the analyzed muscles was 3.76 [3.64]% (median [interquartile range]), and the average water T2 was 40.5 [6.4] ms (Additional file 1: Supplemental Table S4). Both aTSC of the DA-3D-RAD-C and Cartesian GRE acquisitions showed a modest yet statistically significant correlation with PDFF (Spearman rho = 0.357, *p* < 0.001 and Spearman rho = 0.359, *p* < 0.001, respectively). Similarly, both DA-3D-RAD-C and Cartesian GRE acquisitions also showed a significant correlation with the water T2 (Spearman rho = 0.472, *p* < 0.001 and Spearman rho = 0.467, *p* < 0.001, respectively), with a steeper slope observed for the GRE data compared to DA-3D-RAD-C.

## Discussion

At present, there is no standardized approach for both the acquisition and quantification of ^23^Na MRI data, but such standardization is imperative for enabling meaningful comparisons and the broader dissemination of this method. Quantification of the ^23^Na signal highly depends on the acquisition scheme as well as on used references for signal calibration. Therefore, we aimed to investigate the effects of these variations using a large dataset of ^23^Na images of the lower leg.

In a prior study involving patients with paramyotonia congenita, two distinct sequences (a radial UTE with a TE of 0.6 ms and a fast low-angle shot sequence with a TE of 3.53 ms) were employed to measure changes in aTSC subsequent to cold-induced muscle weakness [23]. Following exercise in the cooled leg, the radial UTE signal exhibited a 22% increase, whereas the signal only showed an 8% increase. In addition, the radial sequence also measured sodium accumulation in the non-cooled muscles (second lower leg), suggesting that exercise stress might have contributed to sodium accumulation in these non-cooled muscles. These first observations in patients with muscle weakness suggested a higher sensitivity of the radial UTE in capturing subtle pathological changes in muscle tissue.

Regarding the technical aspects of sodium quantification in skeletal muscle tissue, the short relaxation times of ^23^Na in skeletal muscle tissue significantly impacts the accuracy of sodium quantification. In muscle tissue, the rapid-decaying short T2^*^ component necessitates swift data acquisition. Although optimized UTE sequences for ^23^Na MRI are frequently used in more methodical studies that included healthy subjects (see Table S4), in the majority of clinical studies, Cartesian GRE sequences have been used (see Additional file 1: Supplemental Table S5) [1]. However, the phase encoding gradients of the Cartesian GRE limit the achievable echo times, so that these sequences encounter significant T2^*^ weighting. Considering the measured transverse relaxation times of healthy subjects (see Table 3), at a TE of 2.07 ms used in the present study, approximately 15% less signal intensity is captured compared to a TE of 0.3 ms. On the other hand, due to the longer readout duration in this study, the DA-3D-RAD-C sequence demonstrates more pronounced T2^*^ blurring than a Cartesian GRE. Moreover, relaxation may also occur during the excitation pulse [24, 25]. In our case, the DA-3D-RAD-C sequence utilized a rectangular excitation pulse lasting 0.5 ms, while the Cartesian GRE employed a slice-selective pulse, whose duration can be system-dependent and is often inaccessible retrospectively, but is typically longer than 0.5 ms. Furthermore, the specific point spread function (reflecting the voxel bleeding into neighboring voxels) differs between Cartesian GRE and radial UTE sequences.

The adoption of relatively short TRs, aimed at minimizing scan duration, introduces additional T1 weighting in ^23^Na images. Our TR of 100 ms particularly influences the quantification based on NaCl references (T1 ≈ 60 ms), leading to a decreased reference signal due to the signal saturation and potential overestimation or underestimation of the aTSC depending on sequence parameters. To counteract these relaxation effects, a correction was applied in post-processing. However, accurate correction depends on the discrepancy between assumed and actual relaxation times, which remain unknown. Thus, our aTSC determination is linked to the relaxation coefficients of muscle tissue in relation to those of the corresponding reference solutions used for signal calibration (see Table 3).

While relaxation correction demonstrated to be able to mitigate the impact of distinct relaxation behaviors observed in reference phantoms, it appears inadequate in fully correcting the inherent relaxation behavior specific to muscle tissue. This discrepancy is highlighted by the high correlation observed between aTSC measured using NaCl or agarose for both sequences, where the fitted slopes closely, but not completely approach one. Furthermore, the fitted slope of the correlation curve between DA-3D-RAD-C and Cartesian GRE still exceeded 1. These observations might be explained by different relaxation times within muscle, which could potentially vary among patients and across different pathological conditions.

The remaining offset between the different methods could be also related to field inhomogeneities (Additional file 1: Supplemental Fig. S2). Previously acquired resonance offset maps show higher off-resonances (|> 100 Hz|) in the reference phantoms, which can lead to additional signal blurring. Furthermore, the measured spin-density weighted ^23^Na image depends also on the transmission and reception fields (B_1_^+^ and B_1_^−^, respectively). Our birdcage coil exhibits a B_1_^+^ profile with increased flip angle at the corner, which particularly affects the reference tubes. A template-based B_1_^+^/B_1_^−^ correction approach (as reported in literature [15, 26, 27]) might be used to reduce biases in TSC measurements to avoid additional scan time of individual B_1_^+^/B_1_^−^ measurements.

Low aTSC are frequently related to a higher fat fraction within the muscle tissue, as subcutaneous fat shows lower aTSC compared to normal-appearing muscle tissue [2]. Conversely, higher aTSC values might point to the existence of muscular edema, which could in return lead to longer relaxation times compared to instances where edema is absent. However, it is important to note that literature investigating the influence of pathological processes on ^23^Na relaxation times is limited [28, 29]. Additional research is needed to comprehensively understand and characterize how various pathological conditions may affect relaxation times in skeletal muscle tissue.

Ideally, additional measurements of ^23^Na T2^*^ for each subject could provide personalized relaxation correction, especially in the context of pathological conditions. However, the implementation of such a protocol demands a very fast scanning procedure, a feat that is currently not feasible with established protocols, which require at least 30 min for completion [28, 30–34]. The impracticality of such lengthy protocols in clinical settings underscores the need for further advancements in imaging methodologies to enable rapid acquisition of ^23^Na T2^*^ data without compromising image quality or precision.

The reduced aTSC noted in the tibialis anterior muscle might be partly attributed to a substantial impact of residual quadrupolar interaction of the sodium spin of 3/2. Different pennation angles observed in various lower leg muscles lead to different fiber angles relative to the main magnetic field *B*_0_ and consequently a stronger impact of quadrupolar interaction in tibialis anterior than other muscles. This effect generally results in a faster signal decay of the short component, which could cause an underestimation of aTSC when compared to other muscles. Similar influences have already been described for the determination of the apparent tissue potassium concentration using ^39^K MRI [35].

Regarding the inclusion criteria for analysis, the focus was directed toward technical aspects rather than therapeutic considerations. This approach aimed to emphasize and prioritize the technical components and methodologies employed in the study, providing a big pool of ^23^Na data regardless the underlying pathologies. As data was also analyzed retrospectively, we had no influence on the acquisition parameters, which however are aligned with commonly reported values in existing literature.

A limitation of this work is the calculation of the PDFF maps. As phase images of the Dixon sequence were not stored, PDFF maps were used that were calculated automatically after data acquisition. However, the lack of specification from the vendor regarding the used algorithm in their working package renders the calculation of the provided PDFF maps non-traceable, which introduces some uncertainties in the analysis.

In conclusion, our investigation of ^23^Na MRI methods showed the need of utilizing UTE sequences to mitigate signal loss during quantification. In the absence of a UTE sequence, applying relaxation correction can help address relaxation weighting effects. Despite achieving a considerable correlation post-relaxation correction between Cartesian GRE and DA-3D-RAD-C sequences, individual relaxation time determination may aid further improvement, especially in diseased tissue; however, the extended acquisition times for ^23^Na relaxometry limit its clinical feasibility. Nonetheless, employing relaxation correction is crucial to minimize sequence parameter impacts, and considering additional fat correction is advisable for patients with increased fat fractions.

### Supplementary Information

**Additional file 1:** **Supplemental Table S1.** A summary of ^23^Na MRI studies performed on extremities in healthy subjects. **Supplemental Table S2.** A summary of ^23^Na MRI studies performed on extremities in patient cohorts. **Supplemental Table S3.** Acquisition parameters for ^23^Na relaxometry at a 3-T MRI system. **Supplemental Table S4.** Apparent tissue sodium concentration (aTSC) values measured in lower leg muscles (*n* = 1,477) using the Cartesian GRE and DA-3D-RAD-C sequences, and calibrating the ^23^Na MR signal either using NaCl and agarose (Ag) reference phantoms. Without performing a relaxation correction, aTSC values differed between the two sequences and reference types. After correction of relaxation biases, measured aTSC values were in a similar range. **Supplemental Table S5.** Fat fraction (*n* = 1,447) and water T2 (*n* = 1,371) in lower leg muscles. **Supplemental Fig. S1.** Overlaid reference and muscle masks on ^23^Na images. **Supplemental Fig. S2.** ^23^Na images acquired with a DA-3D-RAD-C sequence (upper row) and the corresponding resonance offset maps (ΔB_0_, middle row) and map of relative effective flip angle (ΔB_1_, lower row) of four different individuals. Supplementary Material.

## Data Availability

The datasets used during the current study are available from the corresponding author on reasonable request.

## Abbreviations

3D: Three-dimensional
aTSC: Apparent tissue sodium concentration
DA-3D-RAD-C: Density-adapted 3D radial readout sequence with cuboid field-of-view
GRE: Gradient-echo
MESE: Multi-echo spin-echo
MRI: Magnetic resonance imaging
PDFF: Proton density fat fraction
ROI: Region of interest
TE: Echo time
TR: Repetition time
Tukey-HSD: Tukey’s honestly significant difference
UTE: Ultra-short echo time
WSRT: Wilcoxon signed rank test
