# Supplementary material for: Sodium quantification in skeletal muscle: comparison between Cartesian gradient-echo and radial ultra-short echo time 23Na MRI techniques
Source: Eur Radiol Exp. 2024 May 22;8:61. doi: 10.1186/s41747-024-00461-1 (PMC11109078; doi:10.1186/s41747-024-00461-1)
Supplement: Supplementary file 1 — Additional file 1: Supplemental Table S1. A summary of 23Na MRI studies performed on extremities in healthy subjects. Supplemental Table S2. A summary of 23Na MRI studies performed on extremities in patient cohorts. Supplemental Table S3. Acquisition parameters for 23Na relaxometry at a 3-T MRI system. Supplemental Table S4. Apparent tissue sodium concentration (aTSC) values measured in lower leg muscles (n = 1,477) using the Cartesian GRE and DA-3D-RAD-C sequences, and calibrating the 23Na MR signal either using NaCl and agarose (Ag) reference phantoms. Without performing a relaxation correction, aTSC values differed between the two sequences and reference types. After correction of relaxation biases, measured aTSC values were in a similar range. Supplemental Table S5. Fat fraction (n = 1,447) and water T2 (n = 1,371) in lower leg muscles. Supplemental Fig. S1. Overlaid reference and muscle masks on 23Na images. Supplemental Fig. S2. 23Na images acquired with a DA-3D-RAD-C sequence (upper row) and the corresponding resonance offset maps (ΔB0, middle row) and map of relative effective flip angle (ΔB1, lower row) of four different individuals. Supplementary Material. [file 41747_2024_461_MOESM1_ESM.pdf]

Sodium quantification in skeletal muscle: comparison between Cartesian gradient-echo and radial ultra-short echo time 23Na MRI techniques

ELECTRONIC SUPPLEMENTARY MATERIAL

Literature research on ^{23}Na MRI sequences in muscle/knee imaging

Supplemental Table S1: A summary of ^{23}Na MRI studies performed on extremities in healthy subjects. Given are the results of muscle or cartilage ^{23}Na MRI studies investigating the repeatability/reproducibility of aTSC determination and/or methodical studies, which were published within the last ten years (searching in PubMed on 06/03/2024). In addition, the vendor, sequence type together with relevant sequence parameters, and potentially applied signal corrections are summarized. For signal calibration all studies used external reference tubes.

Region	Paper	Field Strength	Vendor	Sequence	Number of subjects	TE/TR (ms)	Signal correction	Tissue type	aTSC (mM) [1]
Healthy Muscle	Linz et al. 2015	7T	Siemens	2D-GRE	17 (measured three to five times)	2.27/135	B_1	Skin	30-60 (depending on age)
	Dyke et al. 2018	3T	Siemens	2D-GRE	30 (all measured three times)	1.91/100	None	GM	16.5 ±3.8
								SOL	13.7 ± 2.0
								TA	16.1 ± 3.4
	Gerhalter et al. 2019	3T	Siemens	DA-3D-RAD	8 (all measured three times)	0.3/120	B_0	GM	16.8 ± 2.2
								SOL	17.9 ± 1.5
								TA	14.1 ± 1.2
	Lott et al. 2019	7T	Siemens	DA-3D-RAD	4 (measured once)	0.3/150	?	Posterior muscles	20 ± 3
	Alhulail et al.2021	3T	Siemens	2D-FID UTE MRSI and DA-3D-RAD	4 (measured once)	0.55/650 and 0.3/100	B_0	GM	25.0 ± 2.1
								SOL	34.1 ± 2.2
								TA	25.3 ± 2.1
	Zaric et al. 2022	7T	Siemens	DA-3D-RAD	10 (all measured three times)	0.55/100	B_0/B_1, relax., PVC	GM	19.9 ± 0.1
								SOL	12.6 ± 0.2
								TA	13.8 ± 0.2
	Gast et al. 2022	7T	Siemens	Stack-of-Stars	14 (measured once)	0.3/120	Relax., PVC	GM	18.1 ± 3.1
								SOL	19.0 ± 2.1
								TA	16.7 ± 2.2
	Vaeggemose et al. 2022	3T	GE	DA-3D-RAD	5 (all measured two times)	0.31/5.8	B_1	Thigh muscles	Not given
	Baron et al. 2023	3T	Siemens	DA-3D-RAD	2 (measured four times)	0.2/120	B_1, T_1 corrected	GM	22.2
								SOL	18.2
								TA	14.6
Healthy Cartilage	De Bruin et al. 2015	7T	Phillips	Stack-of-Spiral	7 healthy and 1 patient with osteoarthritis	1/135	None		Not given
	Kamp et al. 2021	3T	Siemens	DA-3D-RAD	10 (protocol 1), 9 (protocol 2)	0.3/70 (protocol 1), 0.3/84 (protocol 2)	Relax., PVC	Patellar cartilage	215 ± 44 (protocol 1), 200 ± 48 (protocol 2)
	Müller-Lutz et al. 2021	3T	Siemens	DA-3D-RAD	10 (scanned with two protocols)	0.3/80 (protocol 1), 0.3/60 (protocol 2)	Relax., PVC	Radiocarpal joint	158 ± 28 (protocol 1)
								Midcarpal joint	173 ± 36 (protocol 1)

DA-3D-RAD density-adapted 3D radial sequence, *GM* gastrocnemius medialis muscle, *GRE* Cartesian gradient echo sequence, *PVC* partial volume correction, *Relax* relaxation correction, *SOL* soleus muscle, *TA* tibialis anterior muscle

[1] only a selection of aTSC values is provided in this table. Note that some papers reported more aTSC values other aTSC values for additional region of interests

Supplemental Table S2. A summary of ^{23}Na MRI studies performed on extremities in patient cohorts. Given are the results of muscle ^{23}Na MRI studies investigating the pathological changes or changes in the sodium signal after intervention, which were published within the last ten years (searching in PubMed on 06/03/2024). In addition, the vendor, sequence type together with relevant sequence parameters, and potentially applied signal corrections are summarized. For signal calibration all studies used external reference tubes, except two cartilage studies, which used no external reference (marked with *).

Intervention / Pathology	Paper	Field Strength	Vendor	^{23}Na Sequence	Subjects	TE/TR (ms)	Signal correction	TSC (mM) or SNR in diseased muscle/cartilage [1]	TSC (mM) in control muscle/cartilage [1]
Exercise/ muscle unloading	Hammon et al. 2015	3T	Siemens	2D-GRE	6 healthy before/ after aerobic/anaerobic exercise	2.07/ 100	None		*Whole leg*<br>Before anaerobic: 34.0 ± 2.2<br>After: 36.2 ± 1.8
	Dahlmann et al. 2016	3T	Siemens	2D-GRE	1 patient with muscle injury (sport-related)	2.07/ 100	None	*Injured area*<br>Day 0: 43.5<br>Week 2: 37.5	
	Gerlach et al. 2017	3T	Siemens	2D-GRE	7 healthy wearing orthoses for 60 days	1.91/ 100	None		*GM*<br>Baseline: 11.6 ± 2.2<br>Days 60: 13.5 ± 2.4
	Hoger et al. 2022	3T	Siemens	DA-3D-RAD	14 healthy before/ after exercise	0.3/ 120	None		*GM*<br>Baseline: 14.3 ± 2.9<br>After: 19.5 ± 2.5
	Gast et al. 2022	7T	Siemens	Stack of Stars	14 healthy before/ after exercise	0.3/ 120	Relax., PVC		*GM*<br>Baseline: 17.7 ± 2.9<br>After: 27.9 ± 4.4
	Petter et al. 2022	3T	Philips	2D-GRE	6 patients with chronic fatigue syndrome and 6 controls before/ after exercise	2.44-39.04/100	None	*GM*<br>Baseline: 10.5 ± 0.8<br>After 1 min: 13.7 ± 0.7<br>After 40 min: 11.7 ± 1.3	*GM*<br>Baseline: 10.2 ± 1.2<br>After 1 min: 12.7 ± 1.3<br>After 40 min: 10.8 ± 1.1
Muscular dystrophy (MD)	Glemser et al. 2017	3T	Siemens	DA-3D-RAD	2 patients with Duchenne MD	0.3/ 120	None	*SOL at baseline*<br>Patient 1: 37<br>Patient 2: 43.2	
	Gerhalter et al. 2019	3T	Siemens	DA-3D-RAD	13 patients with Duchenne MD and 14 controls	0.3/50	Fat fraction	All leg muscles: 26.0 ± 1.3	All leg muscles: 16.5 ± 1.3
	Schüssler et al. 2020	3T	Siemens	DA-3D-RAD	1 patient with Becker MD (baseline, follow up) and 1 control	0.3/50	Fat fraction	*Baseline / 6 month*<br>GM: 18.5 / 18.7	GM: 19.7
	Gerhalter et al. 2021	3T	Siemens	DA-3D-RAD	19 patients with Facioscapulo-humeral MD and 12 controls	0.3/50	Fat fraction	GM: 24.4 (15.9-41.2<br>TP: 15.7 (13.7-19.7)	GM: 20.3 (18.3-21.9)<br>TP: 17.6 (17.1-20.4)
Channelo-pathies with periodic paralysis (PP)	Fan et al. 2013	3T	Siemens	Not specified	1 patient with normokalaemic PP	Not specified	None	*SNR*<br>Left leg: 1.32<br>Right leg: 1.26	
	Weber et al. 2016	7T	Siemens	DA-3D-RAD	5/8 patients with severe/mild hypokalemic PP (HypoPP), and 16 controls	0.35/ 160	B_0, B_1	*All leg muscles*<br>Severe HypoPP: 35.3 ± 9.2<br>Mild HypoPP: 33.0 ± 3.9	19.9 ± 1.9
Cartilage	Widhalm et al. 2016 *	7T	Siemens	3D-GRE	9 patients with patellar dislocation and 9 controls	8.34/ 17	None	*SNR*[†]<br>Medial: 13.6<br>Intermediary: 14.2	*SNR*[†]<br>Medial: 12.1<br>Intermediary: 13.3

	Madelin et al. 2018	7T	Siemens	3D-RAD	12 patients with knee osteoarthritis (baseline and follow-up)	0.4/ 100	Receive sensitivity	*Δ(baseline-follow-up)* Patellar: 29.5 ± 44.9	
	Zbýň et al. 2015	7T	Siemens	3D-GRE	6 MFX patients, 6 MACT patients and 9 controls	8.34/ 17	Receive sensitivity, relax.	*Talar repaired tissue* MFX: 243 ± 58 MACT: 193 ± 33	*Talar cartilage* 320 ± 42
	Zbýň et al. 2020 *	7T	Siemens	3D-GRE	13 patients with chondral lesions	1.22/ 9.2	Receive sensitivity, PVC	*SNR*[††] Lesion: 155.1	*SNR*[††] Healthy weight bearing: 186.8
Systemic disease (e.g. hemodialysis (HD) patients, chronic kidney disease (CKD), obesity…)	Dahlmann et al. 2015	3T	Siemens	2D-GRE	24 HD patients and 27 controls	2.07/ 100	None		Values not available
	Hammon et al. 2015	3T	Siemens	2D-GRE	9 patients with acute heart failure before/ after treatment and 9 controls	2.07/ 100	None	*Before therapy* 30.7 ± 6.4 *After* 24.2 ± 6.1	18.3 ± 2.5
	Hammon et al. 2017	3T	Siemens	2D-GRE	6 patients with kidney injury before/after HD and 14 controls	2.07/ 100	None	*Before HD* 32.7 ± 6.9 *After HD* 31.7 ± 10.2	16.6 ± 2.1
	Kopp et al. 2018	3T	Siemens	2D-GRE	14 patients with diabetes and 30 without before/ after HD	2.07/ 100	None	*Diabetic HD* 27.8 ± 6.1 *Change after HD* -8.3 ± 3.4	*Control HD* 21.5 ± 4.5 *Change after HD* -4.6 ± 3.6
	Crescenzi et al. 2018	3T	Philips	3D-GRE	10 patients with lipedema and 11 controls	0.99/ 130	None	17.5 ± 3.0	14.7 ± 0.8
	Christa et al. 2019	3T	Siemens	3D-GRE	8 patients with hyper-aldosteronism and 12 controls	2.07/ 100	Receive sensitivity	*Relative SNR* 0.19 (0.16-0.22)	*Relative SNR* 0.14 (0.13-0.15)
	Crescenzi et al. 2020	3T	Philips	3D-GRE	15 patients with lipedema and 14 controls	0.99/ 130	None	*Calf* 20.3 ± 3.0	*Calf* 18.3 ± 1.7
	Carranza-Leon et al. 2020	3T	Philips	2D-GRE	23 patients with lupus erythematosus and 28 controls	2.07/ 100	None	18.8 (16.7-18.3)	15.8 (14.7-18.3)
	Qirjazi et al. 2020	3T	GE	2D-GRE	12 patients with CKD, 13 HD, 10 peritoneal dialysis (PD), and 10 controls	1.2/ 100	None	*SOL* CKD: 27.0 ± 5.6 HD: 28.1 ± 6.0 PD: 30.2 ± 6.1	*SOL* 22.0 ± 3.5
	Kannenkeril et al. 2021	3T	Siemens	2D-GRE	52 patients with type 2 Diabetes	2.07/ 100	None	Values not available	
	Puder et al. 2021	3T	Philips	2D-GRE	11 patients with genic obesity (POMC and MC4R) and 12 controls	2.13/ 100	None	POMC: 14.8 (13-24.5) MC4R: 16.4 (14.3-28.7)	11.5 (10.5-12.82)
	Ruggeri Barbaro et al. 2021	3T	Philips	2D-GRE	70 pre-hypertensive subjects	2.07/ 100	None	With high-skin sodium: 17.05 ± 2.9	
	Moosmann et al. 2021	3T	Siemens	2D-GRE	1 patient with enteropathy before/ after treatment and 8 controls	2.07/ 100	None	*Before* 24.7 *After 29 days* 21.7	15.8 ± 1.6
	Dahlmann et al. 2021	3T	Siemens	2D-GRE	31 patients with CKD before/ after treatment and 31 controls	2.07/ 100	None	*Before transplantation* 20.7 ± 5.0 *After 3 months* 16.8 ± 3.0	15.5 ± 1.8
	Filler et al. 2021	3T	GE	2D-GRE	1 patient with Fanconi syndrome	1.2/ 100	None	14.6	
	Filler et al. 2022	3T	GE	2D-GRE	1 patient with hypo-phosphatemic rickets	1.2/ 100	None	21.2	
		3T	Siemens				None	*HD without CVD*	

Systemic disease (e.g. hemodialysis (HD) patients, chronic kidney disease (CKD), obesity…)	Friedrich et al. 2022			2D-GRE	23 HD patients with or 29 without cardiovascular disease	2.07/ 100		21.5 ± 3.6<br>*HD without CVD*<br>24.7 ± 6.0	
	Salerno et al. 2022	3T	GE	DA-2D-RAD	19 patients with CKD, and 17 controls	1.2/ 100	None	19.8 ± 6.5	18.5 ± 1.8
	Christa et al. 2022	3T	Siemens	3D-GRE	8 patients with hyper-aldosteronism before/ after treatment and 12 controls	2.07/ 100	Receive sensitivity	*SNR at baseline*<br>0.19 ± 0.03<br>*At 4 months*<br>0.14 ± 0.02	*SNR*<br>0.14 ± 0.01
	Chifu et al. 2022	3T	Siemens	3D-GRE	8 patients with adrenal insufficiency before/ after treatment and 8 controls	2.07/ 100	None	*SNR*<br>0.15 (0.8-0.18)<br>*After*<br>0.18 (0.14-0.27)	*SNR*<br>0.16 (0.14-0.20)
	Guenes-Altan et al. 2023	3T	Siemens	2D-GRE	58 patients with resistant hypertension before/ after treatment	2.07/ 100	None	*Before treatment*<br>20.6 ± 4.3<br>*After 6 months*<br>20.8± 4.0	
	Ertuglu et al. 2023	3T	Philips	3D-GRE	30 obese patients and 53 controls	0.99/ 130	None	17.1 (15.6-18.7)	15.8 (14.8-19)
	Kolwelter et al. 2023	3T	Siemens	2D-GRE	74 patients with chronic heart failure	2.07/ 100	None	only changes available for muscle	

DA-3D-RAD density-adapted 3D radial sequence, *GM* gastrocnemius medialis muscle, *GRE* Cartesian gradient echo sequence, *PVC* partial volume correction, *Relax* relaxation correction, *SNR* signal to noise ratio, *SOL* soleus muscle, *TA* tibialis anterior muscle, *TP* tibialis posterior muscle

[1] only a selection of aTSC values is provided in this table. Note that some papers reported other aTSC or SNR values for additional region of interests

Supplemental Table S3. Acquisition parameters for ^{23}Na relaxometry at a 3-T MRI system. ^{23}Na signals were acquired with a stack-of-stars sequence with ultra-short echo time (UTE).

Relaxometry	T2* mapping with UTE sequences	T1 mapping with UTE sequences
Specification	4 stack-of-stars sequences with different echo times	8 stack-of-stars sequences with different repetition times
Echo time (ms)	59 TEs: 0.3–70	0.3
Repetition time (ms)	82	11, 20, 30, 40, 60, 80, 120, 300
Flip angle (°)	88	90
Nominal resolution (mm)	4 x 4 x 20	4 x 4 x 20
Readout duration (ms)	2	2
Projections	1910	1910
Averages	1	1
Number of slices	10	10
Acquisition time (min:s)	4 x 2:37<br>Total: 10:28	0:21, 0:38, 0:57, 1:16, 1:55, 2:33, 3:49, 6:22<br>Total: 17: 51

aTSC before and after relaxation correction

Supplemental Table S4: Apparent tissue sodium concentration (aTSC) values measured in lower leg muscles (*n* = 1,477) using the Cartesian GRE and DA-3D-RAD-C sequences, and calibrating the ^{23}Na MR signal either using NaCl and agarose (Ag) reference phantoms. Without performing a relaxation correction, aTSC values differed between the two sequences and reference types. After correction of relaxation biases, measured aTSC values were in a similar range.

	2D Cartesian GRE				DA-3D-RAD-C			
aTSC (mM)	uncorrected		relaxation corrected		uncorrected		relaxation corrected	
	NaCl	Ag	NaCl	Ag	NaCl	Ag	NaCl	Ag
**Mean**	17.30	19.86	20.10	19.48	23.12	19.55	20.6	19.62
**Median**	16.74	19.17	19.50	18.82	22.52	19.07	20.05	19.14
**Standard deviation**	4.48	5.31	4.96	5.10	4.57	4.030	4.05	4.03
**Variance**	20.08	28.16	24.65	25.99	20.91	16.27	16.37	16.22
**25 percentiles**	14.18	16.13	16.64	15.88	19.84	16.71	17.69	16.78
**75 percentiles**	19.98	23.04	23.09	22.54	25.68	21.89	22.87	21.95

Fat fraction and water T2 in skeletal muscle tissue

Supplemental Table S5: **Fat fraction (*n* = 1,447) and water T2 (*n* = 1,371) in lower leg muscles.**

	fat fraction (%)	water T2 (ms)
**Mean**	5.45	40.88
**Median**	3.76	40.46
**Standard deviation**	6.51	4.97
**Variance**	42.44	24.73
**25 percentiles**	2.26	37.53
**75 percentiles**	3.76	40.46

ROI masking of references and individual muscles

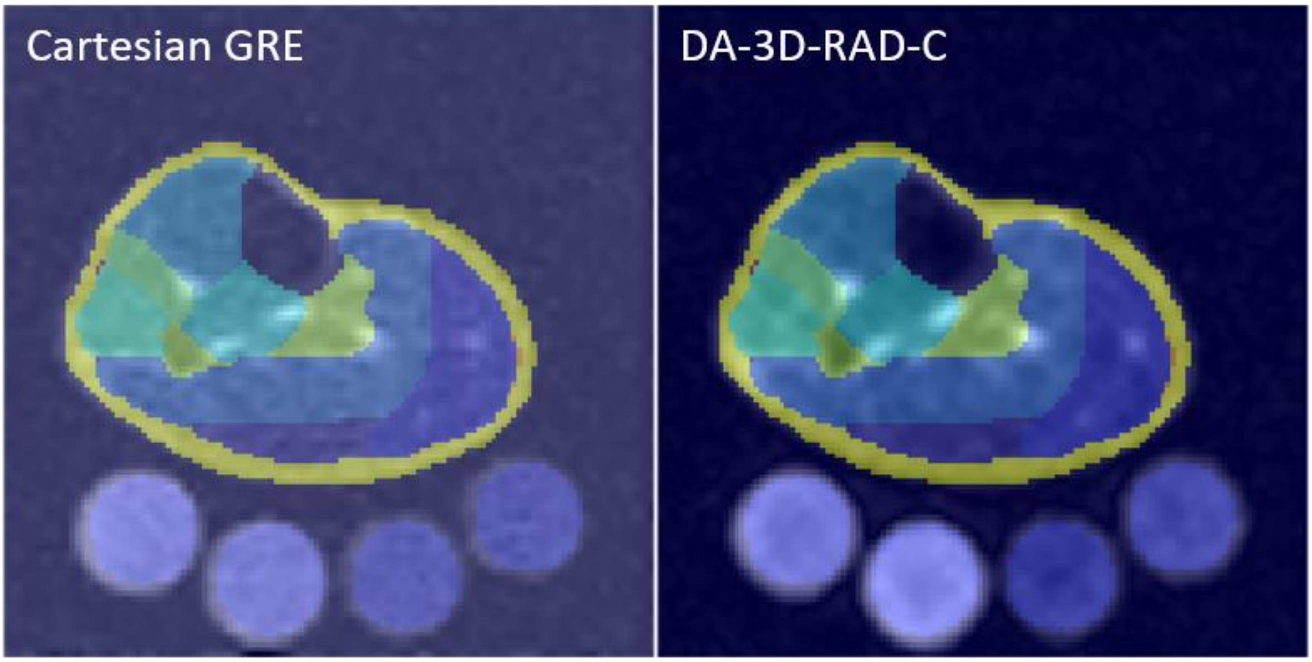

Supplemental Fig. S1. Overlaid reference and muscle masks on ^{23}Na images. Individual muscles (in shaded of blue and green) were semi-automatically segmented on a ^{1}H-Dixon images. Subcutaneous fat and left-over muscle/blood areas are highlighted in yellow. Reference masks were segmented once manually in MITK (www.mitk.org) to create a reference template that was then co-registered to each ^{23}Na MRI data set. The co-registered reference masks were then cropped by one pixel before the calculation of the mean signal intensity value within the individual reference ROIs.

In-vivo B_1 and B_0 maps of ^{23}Na knee coil

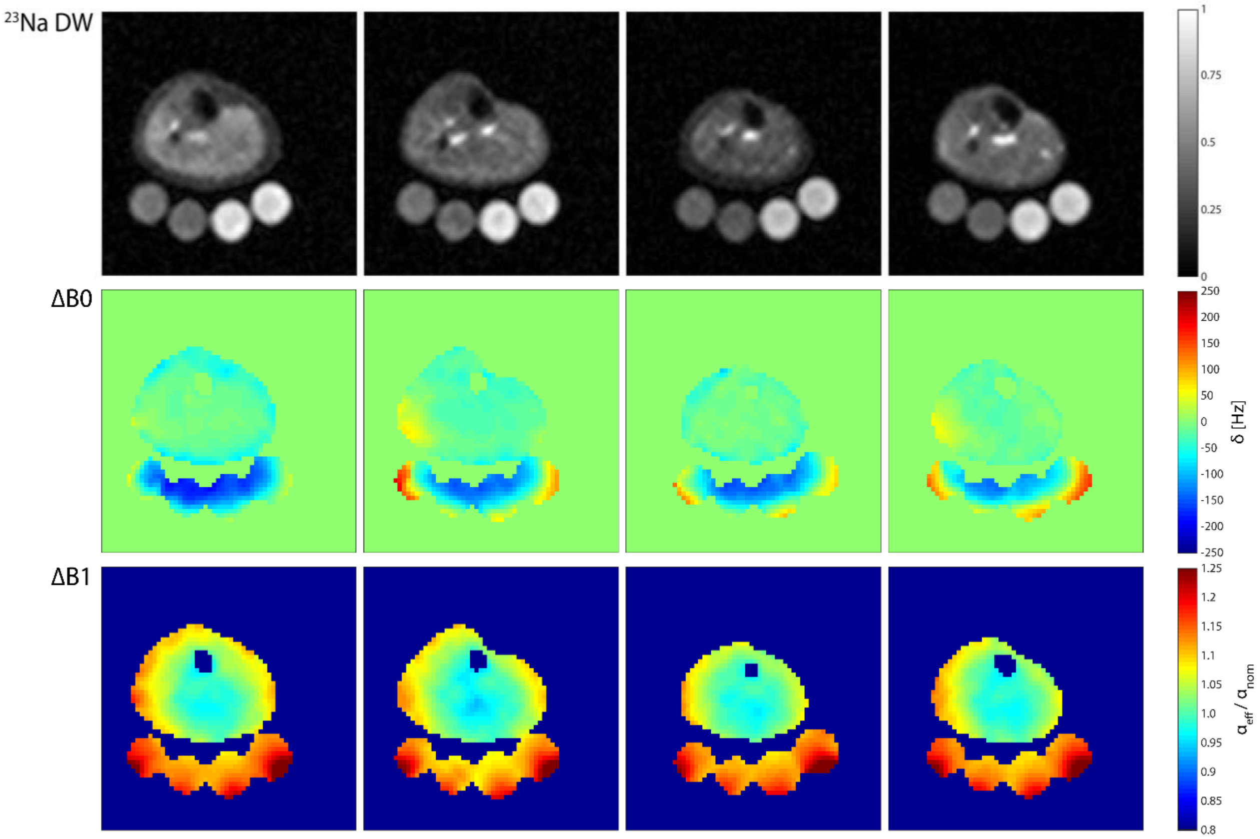

Supplemental Fig. S2. ^{23}Na images acquired with a DA-3D-RAD-C sequence (upper row) and the corresponding resonance offset maps (ΔB0, middle row) and map of relative effective flip angle (ΔB1, lower row) of four different individuals. Acquisition parameters and data processing steps are described in Gast et al. (NMR in Biomedicine 2018 DOI: 10.1002/nbm.4010).

Supplementary Material

Relaxometry at 3-T

To determine T1 and T2* for relaxation correction, ^{23}Na relaxometry was performed at 3-T (Vida, Siemens Healthineers, Erlangen, Germany) with the ^{23}Na knee coil (Stark Contrast, Germany) in 14 healthy volunteers (aged 36.9 ± 8.1 years). Acquisition parameters are listed in **Supplemental Table S1**. Region of interests (ROIs) were drawn manually on the soleus muscle in the middle slice and the reference phantoms. Care was taken to exclude any blood vessels when placing the ROI within the muscle tissue. The relaxation times were calculated based on the mean intensity of ROIs to ensure a stable fit. The T1 signal recovery was fitted according to

$$S_{signal\ recovery}(TR) = M_0\left(1 - e^{-\frac{TR}{T1}}\right).$$

For agarose references and muscle, a biexponential T2* signal decay was fitted according to

$$S_{biexponential\ decay}(TE) = \sqrt{A^2\left(0.6\, e^{-\frac{TE}{T2_s^*}} + 0.4\, e^{-\frac{TE}{T2_l^*}}\right)^2 + n^2}$$

including the Rician noise-related parameter n. Reference tubes without agarose were fitted to a monoexponential fit

$$S_{monoexponential\ decay}(TE) = \sqrt{A^2\left(e^{-\frac{TE}{T2^*}}\right)^2 + n^2}\ .$$

The calculation of the relaxation times was done in a custom-written MATLAB script using the fit function with a non-linear least squares' method, start points and upper and lower boundaries. We used the ROI of the soleus muscle for all muscle as the relaxation times do not vary a lot between posterior and anterior muscles as previously shown by Zaric et al (2022) and Alhulail et al (2020). This global approach is commonly applied in literature (*e.g.* Lott et al 2019 MRM, Zaric et al 2022, James et al 2015, Meyer et al 2019) instead of a muscle-wise approach with different correction factors per muscle.
